# Use of Different Proteases to Obtain Flaxseed Protein Hydrolysates with Antioxidant Activity

**DOI:** 10.3390/ijms17071027

**Published:** 2016-06-29

**Authors:** Magdalena Karamać, Agnieszka Kosińska-Cagnazzo, Anna Kulczyk

**Affiliations:** 1 Department of Chemical and Physical Properties of Food, Institute of Animal Reproduction and Food Research, Polish Academy of Sciences, Tuwima 10 Str., 10-748 Olsztyn, Poland; agnieszka.kosinska@hevs.ch (A.K.-C.); anna.urbalewicz@gmail.com (A.K.); 2 Institute of Life Technologies, University of Applied Sciences and Arts Western Switzerland, Route du Rawyl 47, 1950 Sion, Switzerland

**Keywords:** flaxseed cake, enzymatic hydrolysis, protein hydrolysates, proteases, antioxidant activity

## Abstract

The antioxidant activity of flaxseed protein hydrolysates obtained using five different enzymes was evaluated. Proteins were isolated from flaxseed cake and were separately treated with papain, trypsin, pancreatin, Alcalase and Flavourzyme. The degree of hydrolysis (DH) was determined as the percentage of cleaved peptide bonds using a spectrophotometric method with *o*-phthaldialdehyde. The distribution of the molecular weights (MW) of the hydrolysis products was profiled using Tricine-sodium dodecyl sulfate-polyacrylamide gel electrophoresis (Tricine-SDS-PAGE) and size exclusion-high performance liquid chromatography (SE-HPLC) separations. The antioxidant activities of the protein isolate and hydrolysates were probed for their radical scavenging activity using 2,2′-azino-bis-(3-ethylbenzothiazoline-6-sulfonate) radical cation (ABTS^•+^) and photochemiluminescence (PCL-ACL) assays, and for their ferric reducing antioxidant power (FRAP) and ability to bind Fe^2+^. The hydrolysates were more effective as antioxidants than the protein isolate in all systems. The PCL-ACL values of the hydrolysates ranged from 7.2 to 35.7 μmol Trolox/g. Both the FRAP and ABTS^•+^ scavenging activity differed among the hydrolysates to a lower extent, with the ranges of 0.20–0.24 mmol Fe^2+^/g and 0.17–0.22 mmol Trolox/g, respectively. The highest chelating activity (71.5%) was noted for the pancreatin hydrolysate. In general, the hydrolysates obtained using Alcalase and pancreatin had the highest antioxidant activity, even though their DH (15.4% and 29.3%, respectively) and the MW profiles of the peptides varied substantially. The O_2_^•−^ scavenging activity and the ability to chelate Fe^2+^ of the Flavourzyme hydrolysate were lower than those of the Alcalase and pancreatin hydrolysates. Papain was the least effective in releasing the peptides with antioxidant activity. The study showed that the type of enzyme used for flaxseed protein hydrolysis determines the antioxidant activity of the hydrolysates.

## 1. Introduction

The oxidation of lipids and other labile components of food products results in a reduction in their nutritional value and sensorial quality, as well as a shorter shelf life [1]. In the food industry, to prevent undesirable changes, antioxidants are applied as additives to limit oxidation. Artificial antioxidants, such as butylated hydroxytoluene (BHT), butylated hydroxyanisole (BHA) and *n*-propyl gallate, are highly effective; however, their application in foodstuffs is strictly regulated and even forbidden in certain countries because of their potential harmful effects on human health [2]. Therefore, the interest in safe antioxidants from natural sources that could replace the artificial ones is on the rise. Antioxidants also play an important role in living organisms. An excess of free radicals in cells may cause destructive effects by oxidizing DNA, membrane lipids and cellular proteins [3]. Oxidative stress, which is an imbalance between oxidative species and endogenous antioxidant systems in the human organism, has been associated with a number of chronic diseases. It can be reduced by exogenous antioxidants found in foods [3]. Food is an abundant source of natural antioxidants, such as ascorbic acid, carotenoids, tocopherols, phenolic compounds, etc. Nevertheless, the intensive search for new antioxidants is still underway. In recent years, an increasing interest in protein-derived peptides as both food preservatives and nutraceuticals has developed [4]. Pihlanto [2] suggested that protein hydrolysates might be used as natural antioxidants in developing functional foods with increased antioxidant activity.

Antioxidant peptides are encrypted in the amino acid sequence of proteins as inactive fragments. Their release might occur because of enzyme-catalysed hydrolysis in vitro, microorganism activity in fermented food, or autolysis and the gastrointestinal digestion of proteins after human consumption [5]. It is well known that there are various factors determining the antioxidant activity of food protein hydrolysates. An important factor is the specificity of the enzymes used for proteolysis [6,7,8] and the degree of hydrolysis (DH) [8,9,10]. The latter depends on the conditions of the process carried out for the specific protease-protein system, such as temperature, pH and time of hydrolysis, the ratio of enzyme to substrate and the initial concentration of the proteins [11,12]. The effectiveness of the hydrolysates as antioxidants is strictly linked to the characteristics of the released peptides, e.g., their molecular weights, amino acid composition and sequence of amino acids [7,13,14,15]. Apart from their chemical composition, the hydrophobicity of peptides might substantially affect the antioxidant activity of hydrolysates [14,16]. In spite of a large number of studies being carried out to determine the correlation between the above-mentioned factors and the antioxidant activity of hydrolysates, the results obtained are still ambiguous. The issue becomes increasingly complex because of the various modes of action of hydrolysates as antioxidants. First of all, hydrolysates are capable of scavenging or quenching free radicals and reactive oxygen species [7,13,16]. They also have significant reducing power [8,17] and can chelate prooxidative metal ions [16,18].

Several proteins of both animal and plant origin were tested as substrates for obtaining enzymatic hydrolysates with antioxidant activity, and among them, there were proteins from flaxseed [2,4,5]. Defatted flaxseeds are rich in proteins, accounting for 31%–50% (*w*/*w*) with a relatively high content of aspartic acid, glutamic acid, leucine and arginine [19,20]. To release the peptides with antioxidant activity encrypted in flaxseed proteins, both commercial proteases (Flavourzyme, Alcalase, papain, ficin, thermolysin, pronase, trypsin, pepsin, pancreatin) [9,10,12,21,22] and crude protease isolated from *Bacillus altitudinis* [18] were used. Four proteolytic microbial strains were also applied for the fermentation of flaxseed flour [23]. In the cited studies, the authors demonstrated the antioxidant activity of flaxseed protein hydrolysates obtained using one enzyme [9,10,12,18] or with a multistep hydrolysis using various proteases to obtain peptide fractions with designed properties, such as a low content of aromatic amino acids [22]. Only one study, which was carried out by Udenigwe et al. [21], reported on the parallel hydrolysis of flaxseed proteins with various enzymes. The authors compared the properties of the peptide fractions with the low molecular weight (MW < 1 kDa) isolated from the hydrolysates and determined their antiradical activity. There is no data in the literature comparing the antioxidant activity of whole hydrolysates obtained with proteases with different specificity and origin (plant, animal, microbiological). Therefore, the aim of our study was to produce flaxseed protein hydrolysates using five proteases and to compare their antioxidant activity in terms of their radical scavenging activity, metal ion chelating activity and reducing power.

## 2. Results and Discussion

### 2.1. Characteristic of Flaxseed Protein Hydrolysates

**Figure 1:**
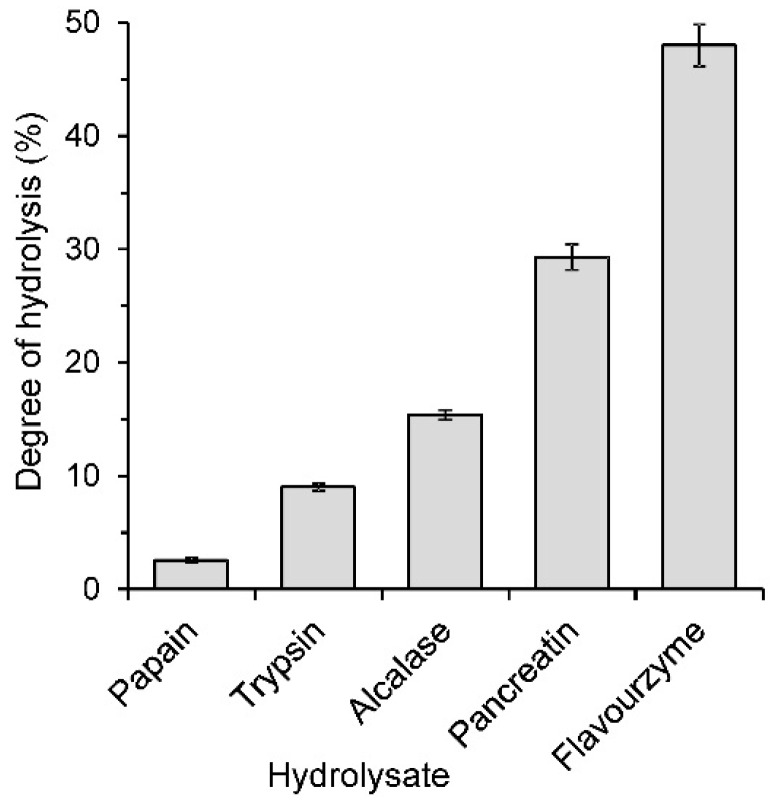
Degree of hydrolysis (DH) of flaxseed protein hydrolysates.

The isolated flaxseed proteins were treated with papain, trypsin, Alcalase, pancreatin or Flavourzyme. To compare the effectiveness of the enzymes, constant parameters were applied, e.g., the temperature (50 °C), the initial concentration of the substrate (5%), the enzyme-to-substrate ratio (E/S, 15 mAU/g) and the reaction time (120 min). The flaxseed protein isolate was most effectively hydrolysed with Flavourzyme (Figure 1). The DH reached 48.0% after 120 min of hydrolysis. A high DH of 29.3% was also noted for the pancreatin hydrolysate. In the case of trypsin and papain hydrolysis, the DH values were substantially lower and did not exceed 10%. Previous attempts for the enzymatic hydrolysis of flaxseed proteins have shown that Flavourzyme might cleave peptide bonds, even up to 70.62% [12]. In turn, the study of Silva et al. [9] showed that at the optimal conditions of the process (180 min, 60 °C, E/S 1:48 and pH 7.8), Alcalase allows the DH to reach 19.3%, a value comparable to that obtained in the present study. Pancreatin was used for flaxseed protein hydrolysis by Marambe et al. [24] at conditions simulating the action of gastrointestinal enzymes. Pancreatin digestion was preceded by pepsin digestion and carried out in the presence of bile salts. The final product was characterised with a DH of 43.95%. The low DH found for papain and trypsin hydrolysates in the present study might be explained by the high substrate specificity of those enzymes. The hydrolysis of other plant proteins (e.g., pea, soya, peanut) with trypsin and papain resulted in the cleavage of only a few percent of peptide bonds [11,25,26].

**Figure 2:**
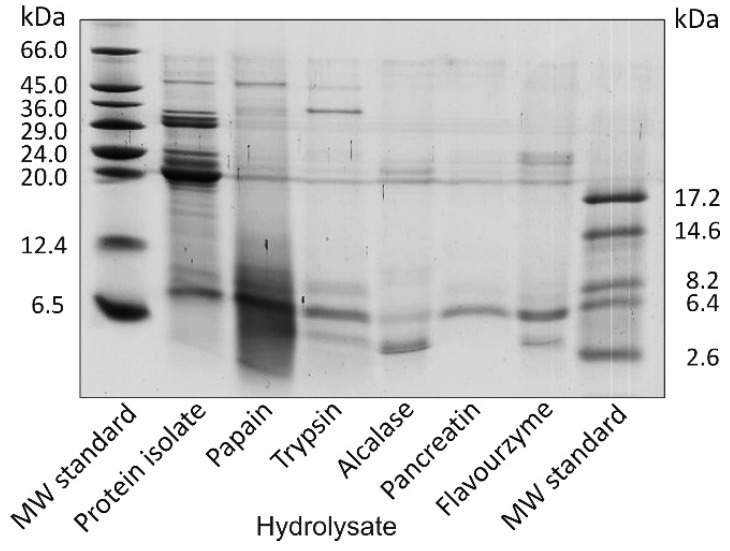
Tricine-sodium dodecyl sulfate-polyacrylamide gel electrophoresis (Tricine-SDS-PAGE) separation of flaxseed protein isolate and its hydrolysates. MW standard: molecular weight standard.

The electropherogram of the Tricine-sodium dodecyl sulfate-polyacrylamide gel electrophoresis (Tricine-SDS-PAGE) separation of the flaxseed protein isolate and hydrolysates is shown in Figure 2. Eight intensively stained bands corresponding to the MWs of 48.5, 33.4, 30.2, 29.2, 24.3, 22.0, 19.5 and 7.9 kDa were visible in the case of the protein isolate. The obtained Tricine-SDS-PAGE pattern was typical for flaxseed protein separation under reducing conditions [9,12]. The bands in the range of the MWs of 19.5–48.5 kDa probably originated from α- and β-chain subunits of 11S globulin [19]. Legumin-like globulins are a predominant fraction of flaxseed proteins and amount to 66% of total proteins [20]. Flaxseed albumins (2S protein) are free from interchain disulfide bonds, and in the SDS-PAGE gel, they were visible as a single band (7–10 kDa) [19,20]. Enzymatic treatment resulted in the hydrolysis of most of the flaxseed proteins, the partial or total disappearance of the corresponding bands, and the appearance of new ones in the lower range of MWs (Figure 2). The bands with a MW higher than 7.9 kDa were not visible in the Tricine-SDS-PAGE separation of pancreatin hydrolysate. In turn, the papain hydrolysate pattern was characterised by the highest number of the bands corresponding to high MWs. Flavourzyme did not completely hydrolyse, and it had protein subunits with MWs of 24.3, 22.0 and 19.5 kDa. This observation is consistent with the earlier study of Marambe et al. [12]. The authors demonstrated the presence of a fraction partially resistant to Flavourzyme in the flaxseed protein isolate. This fraction was visible in the Tricine-SDS-PAGE gel (non-reducing conditions) as 34–43 kDa bands. In turn, Silva et al. [9], on the basis of electrophoretic separation, stated that none of the subunits of flaxseed protein isolates were resistant to hydrolysis by Alcalase. In our study, in the case of the Alcalase hydrolysate, faintly marked bands with MWs of 22.0 and 19.5 kDa were observed, corresponding to the subunits present in the isolate.

**Figure 3:**
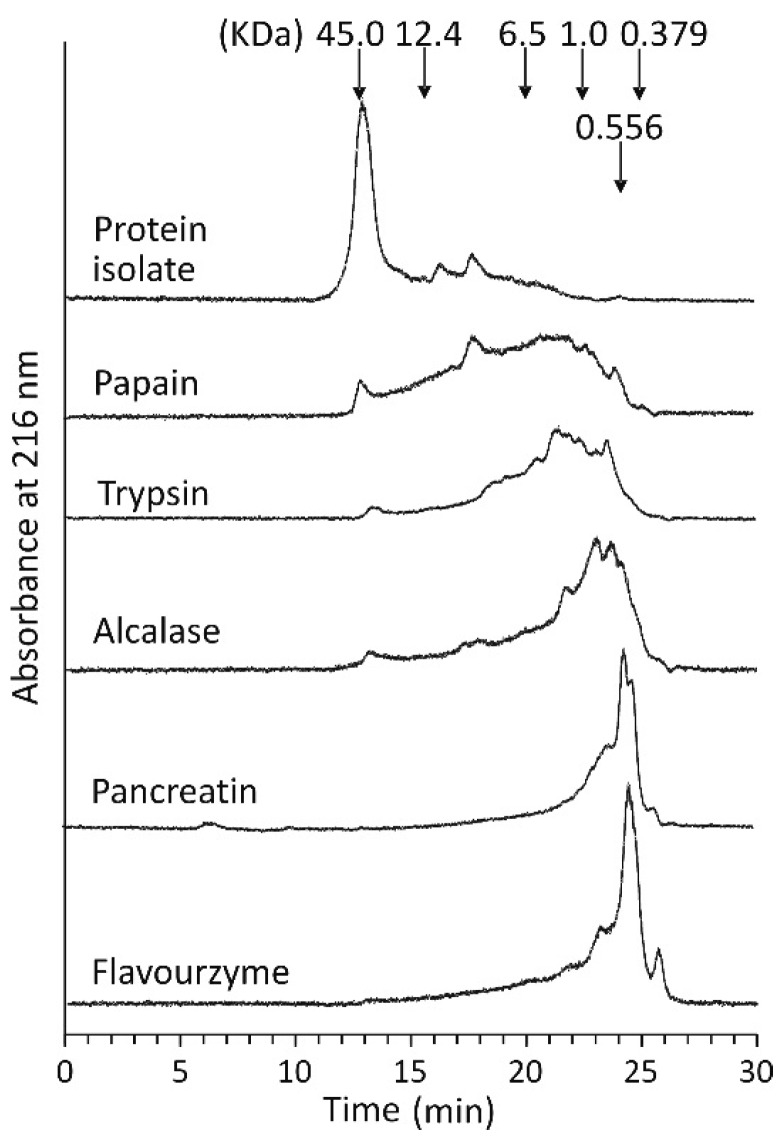
Size exclusion-high performance liquid chromatography (SE-HPLC) chromatograms of flaxseed protein isolate and its hydrolysates.

The MW distributions of the flaxseed protein hydrolysis products obtained using individual enzymes were examined by size exclusion-high performance liquid chromatography (SE-HPLC) separations (Figure 3). The components of the protein isolate were eluted as one high peak with a retention time (*t_r_*) of 12.3 min and several much smaller, broad and poorly separated peaks with longer retention times. The main peak corresponded to the 11S globulin fraction [19]. The low MW proteins and the non-protein ingredients of the isolate absorbing UV light at 216 nm were eluted at longer retention times. The SE-HPLC separation of the hydrolysates revealed that the peak at a *t_r_* of 12.3 min was significantly lower (hydrolysates obtained with papain, trypsin and Alcalase) or not present (pancreatin and Flavourzyme), and at the same time, new peaks corresponding to hydrolysis products appeared.

Based on the calibration curve constructed for polypeptides and peptides with a known MW, it can be concluded that the composition of the hydrolysates obtained with Flavourzyme and pancreatin was comprised mainly of peptides with a MW lower than 1 kDa. In the Flavourzyme hydrolysate, those with a MW of 390 Da were predominant, whereas in the pancreatin hydrolysate, they were slightly larger, at 450 Da. The trypsin and papain hydrolysates contained polypeptides and peptides with a wide range of MWs. In contrast, Alcalase effectively degraded flaxseed protein to the products with a MW < 6.5 kDa. The bacterium *Bacillus subtilis*, which produces Alcalase, was used for the fermentation of the flaxseed protein isolate by Pihlanto et al. [23]. The authors found that 93.1% of the fermentation products represented the fraction with a MW of approximately 5–10 kDa. Thus, the Alcalase hydrolysate in our study contained peptides with lower MWs in comparison to the flaxseed protein isolate treated with *Bacillus subtilis*.

### 2.2. Antioxidant Activity of Flaxseed Protein Hydrolysates

**Figure 4:**
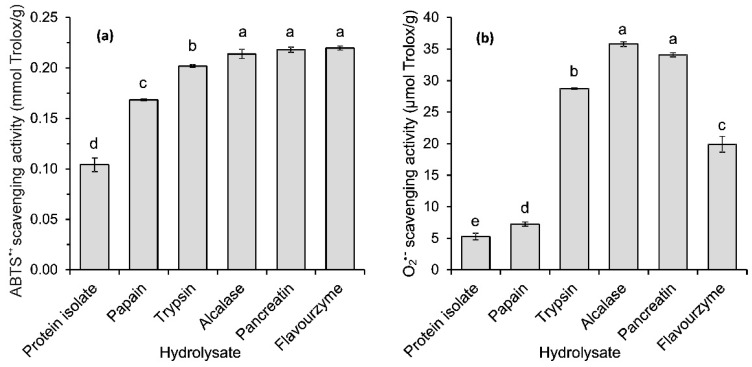
Antiradical activity of flaxseed protein isolate and its hydrolysates towards (**a**) 2,2′- azino-bis-(3-ethylbenzothiazoline-6-sulfonate) radical cations (ABTS^•+^) and (**b**) superoxide radical anions (O_2_^•−^) determined by a photochemiluminescence assay. The bars with different letters (a–e) are significantly different at *p* < 0.05.

The antiradical activity of flaxseed protein hydrolysates was determined towards synthetic 2,2′-azino-bis-(3-ethylbenzothiazoline-6-sulfonate) radical cation (ABTS^•+^) and biologically relevant superoxide radical anion (O_2_^•−^), which was generated from luminol (PCL-ACL) in our study. In both assays, all hydrolysates analysed exhibited higher radical scavenging ability than the flaxseed protein isolate (Figure 4), indicating that the antioxidant peptides were released from the isolate during hydrolysis. The 1.6–2.1-fold and 1.4–6.8-fold differences between the non-hydrolysed and hydrolysed samples were noted in the ABTS and PCL-ACL assays, respectively. When the antiradical activity of the individual hydrolysates was compared, ABTS^•+^ was scavenged to the lowest extent by papain hydrolysate (0.17 mmol Trolox/g) (Figure 4a). The antiradical activity of the trypsin hydrolysate was 0.20 mmol Trolox/g. Higher values were noted for flaxseed protein hydrolysates obtained with the remaining enzymes. The ABTS^•+^ scavenging activities of Alcalase, Flavourzyme and pancreatin hydrolysates were similar (*p* > 0.05), despite significant differences in both the DH and MW distributions of peptides of those hydrolysates (Figure 1 and Figure 3). Some previous studies have shown that the antiradical activity of the hydrolysates towards ABTS^•+^ was related to the DH and MW of the released peptides. The scavenging activity was reported to increase along with the DH [8,10] and with the decreasing MW of the peptides [7,15]. However, it should be noted that these conclusions were made based on the experiments where the hydrolysates or the peptide fractions obtained with only single enzymes were compared. In our study, neither the high DH of the Flavourzyme hydrolysate (Figure 1) nor the predominance of the peptides with a MW < 1 kDa of pancreatin and Flavourzyme hydrolysates (Figure 3) warranted their far greater ABTS^•+^ antiradical activity among the tested samples (Figure 4a). On the other hand, it is worth noting that the Alcalase hydrolysate had relatively high ABTS^•+^ scavenging activity with respect to its DH and MW profiles (Figure 1 and Figure 3). This may suggest that the release of peptides able to scavenge ABTS radical cations depends on the specificity of the proteases used in the hydrolysis of flaxseed proteins and on the amino acid composition of proteins. The higher ABTS^•+^ antiradical activity of the Alcalase hydrolysates in comparison to the activity of the products obtained with other enzymes is confirmed in the literature. Alcalase released peptides from canola meal proteins capable of scavenging ABTS^•+^ more effectively than those obtained with pancreatin, trypsin and pepsin [7]. Additionally, in the case of the protein concentrates of Azufrado beans in the ABTS test, higher values were noted for hydrolysates obtained with Alcalase than for those produced with pancreatin and thermolysin [6]. Tang et al. [13] found that the antiradical activity of the Alcalase hydrolysate of zein resulted from the joint action of small, intermediate and large peptides. Our results suggest that this phenomenon may also take place in the case of flaxseed proteins treated with this enzyme.

The variation in the scavenging activity for superoxide radical anions between the hydrolysates was higher (from 7.2 to 35.7 μmol Trolox/g) than in the case of the antiradical activity against ABTS^•+^ (Figure 4). There was a similar order (papain < trypsin < Alcalase = pancreatin hydrolysate (*p* < 0.05)) of increasing activity in both assays. It should be noted that the Flavourzyme hydrolysate was characterised by an unexpectedly low ability to disable O_2_^•−^. The differences in the scavenging of ABTS^•+^ and O_2_^•−^ by the hydrolysates can be explained by the conditions used in the tests. ABTS^•+^ is water-soluble, and the ABTS assay was carried out in an aqueous environment. In turn, the PCL-ACL assay sample was dissolved, and the reaction was performed in methanol. Therefore, it can be assumed that in the PCL-ACL assay, peptides with a higher hydrophobicity contributed to a higher extent to the activity determined. At the same time, the data of the literature indicate a positive correlation between the O_2_^•−^ scavenging activity and the content of hydrophobic amino acids in hydrolysis products [16,17]. Tang et al. [13] fractionated the zein hydrolysate using reversed phase-high performance liquid chromatography (RP-HPLC) and found that the fraction with the highest hydrophobicity was characterised by the strongest antiradical activity against O_2_^•−^, and ABTS^•+^ was efficiently inactivated by the far more hydrophilic fraction. Li et al. [17] reported that the low MW fraction obtained from chickpea protein hydrolysate which had a high ability to inactivate O_2_^•−^ was high in valine, phenylalanine, isoleucine, leucine and methionine. In turn, on the basis of chemometric analysis, Udenigwe and Aluko [14] showed that the high hydrophobicity of the products of hydrolysis was a strong positive contributor to the scavenging of free radicals, such as 2,2-diphenyl-1-picrylhydrazyl radical (DPPH^•^) and O_2_^•−^. Based on these data, we can speculate that in our study, contrary to the ABTS test, the weaker ability to inactivate O_2_^•−^ of the Flavourzyme hydrolysate compared to the pancreatin and Alcalase hydrolysates could have resulted from its potentially lower hydrophobicity compared to the others.

The O_2_^•−^ scavenging activity of the fractions of Alcalase flaxseed protein hydrolysate was determined by Udenigwe et al. [21]. The authors demonstrated that the fractions of cationic peptides with MW 1–3 and 3–5 kDa were characterised by a higher ability to quench O_2_^•−^. Our findings agree with these observations. In the above-mentioned study, the lack of O_2_^•−^ scavenging activity for a fraction with a MW < 1 kDa obtained from the pancreatin flaxseed protein hydrolysate was noted. In contrast, the results of the present study showed that the pancreatin hydrolysate, which contained mainly peptides with MW < 1 kDa, was the most effective in quenching O_2_^•−^ among the samples tested.

**Figure 5:**
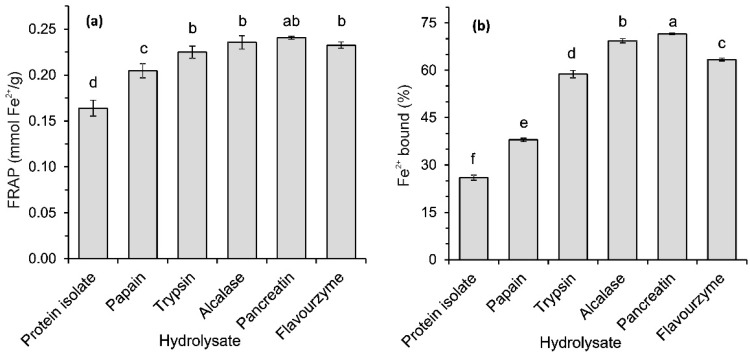
(**a**) Ferric reducing antioxidant activity (FRAP) and (**b**) ability to chelate Fe^2+^ of flaxseed protein isolate and its hydrolysates. The bars with different letters (a–f) are significantly different at *p* < 0.05.

The ferric reducing antioxidant power (FRAP) of the flaxseed protein isolate and its enzymatic hydrolysates is shown in Figure 5a. The hydrolysates, regardless of the type of protease used, acted as reducing agents stronger than the protein isolate (*p* < 0.05). The ability to reduce Fe^3+^ exhibited by trypsin, Alcalase, pancreatin and Flavourzyme hydrolysates was similar and ranged from 0.22 to 0.24 mmol Fe^2+^/g. The lower FRAP value was recorded for papain-treated proteins (*p* < 0.05). Popović et al. [8] observed that Alcalase effectively released peptides capable of reducing Fe^3+^ from pumpkin oil cake globulins, but the hydrolysate of the same proteins obtained with Flavourzyme did not demonstrate significant reducing power. The reducing power of hydrolysates or their fractions was determined in many studies. However, it is difficult to compare the FRAP values obtained in our study with literature data. To determine the reducing power, most studies used the test in which the Fe^3+^/ferricyanide complex was reduced to its ferrous form, and the results are expressed as the absorbance units at 700 nm [16,17,18]. The FRAP measured as the ability to reduce Fe^3+^(TPTZ)_2_ and calculated as equivalents of FeSO_4_ was reported by Silva et al. [9] for flaxseed protein hydrolysates produced with Alcalase. The FRAP ranged from 32.4 to 41.7 mg FeSO_4_/g (depending upon the conditions of hydrolysis). The results obtained in the present study for the Alcalase hydrolysate were similar. Additionally, the ability of pancreatin hydrolysate to reduce ferric ions was comparable to the FRAP values described in our earlier work [10]. It was evident that for the tested hydrolysates, higher values of FRAP correspond to higher values in the ABTS assay (Figure 4a and Figure 5a). This similarity is not surprising if one takes into account the fact that the mechanisms of action of the antioxidants in FRAP and ABTS assays are similar [27]. Antioxidants in both assays react by hydrogen atom transfer, and the redox potential of Fe^3+^-TPTZ and ABTS^•+^ is comparable.

Figure 5b shows the Fe^2+^ chelation activity of the isolate and hydrolysates of flaxseed proteins. The protein isolate at a concentration of 1.54 mg/mL bound 25.9% of the Fe^2+^ introduced into the reaction mixture. The slightly lower metal chelating activity of flaxseed protein showed by Hwang et al. [18] was approximately 15% at a concentration of 2.3 mg/mL. The activities of all the hydrolysates tested were higher than that of the protein isolate (*p* < 0.05) (Figure 5b). Again, as in the previous tests, the type of enzymes used in the process determined the ability of the hydrolysates to chelate ferrous ions. The metal chelating activity of hydrolysates decreased in the following order: pancreatin > Alcalase > Flavourzyme > trypsin > papain. The value observed for the most active hydrolysate (71.5% at a concentration of 1.54 mg/mL) was comparable to the ferrous ions chelation activity noted for the flaxseed proteins treated with the protease from *Bacillus altitudinis* HK02, which was approximately 95% at a concentration of sample of 2.3 mg/mL [18]. Pea protein hydrolysate obtained using thermolysin also chelated 95% of the Fe^2+^ present, but at a lower concentration of 1 mg/mL [16]. Hwang et al. [18] showed that peptides released from flaxseed proteins by the protease from *Bacillus altitudinis* are highly capable of chelating ferrous ions and had a MW < 1 and 1–3 kDa. Fractions with MWs of 3–5 and 5–10 kDa exhibited approximately 10-fold weaker chelating activity, whereas the activity of peptides with MW > 10 kDa was negligible. The correlation of the metal ion chelating activity and the MW of the peptide fractions noted in the cited study do not agree with our observations made for the hydrolysates obtained using various proteases. The Flavourzyme hydrolysate, containing peptides with the lowest MW, chelated ferrous ions to a lesser extent than the pancreatin hydrolysate (MW < 1 kDa) or the Alcalase hydrolysate (MW < 6.5 kDa) (Figure 3 and Figure 5b).

## 3. Materials and Methods

### 3.1. Materials and Reagents

Flaxseed cake was obtained from the Oil Production Plant in Grodzisk Wielkopolski, Poland, as a by-product of oil pressing from flaxseed (*Linum usitatissimum* L.) of the Recital variety.

The enzymes trypsin from porcine pancreas type II-S (EC 3.4.21.4; 1645 BAEE units/mg), papain from papaya latex (EC 3.4.22.2; 12 units/mg protein), pancreatin from porcine pancreas (8 × USP specifications), Alcalase^®^ 2.4 L (EC 3.4.21.62), Flavourzyme^®^ 500 L, and cellulase from *Aspergillus niger* (≈0.8 units/mg) were purchased from Sigma-Aldrich (Saint Louis, MO, USA).

The reagents and standards *o*-phthaldialdehyde (OPA), l-leucine, SE-HPLC standards, glycine, tricine, SDS, acrylamide, *N*,*N*′-methylenebis(acrylamide), Sigma Marker Low Range (MW 6.5–66 kDa) for electrophoresis, [2,2′-azino-bis-(3-ethylbenzothiazoline-6-sulfonic acid)] diammonium salt (ABTS), 6-hydroxy-2,5,7,8-tetramethyl-chroman-2-carboxylic acid (Trolox), ferrozine and 2,4,6-tris(2-pyridyl)-*S*-triazine (TPTZ) were acquired from Sigma-Aldrich. The ACL (antioxidant capacity of lipid soluble substances) kit was purchased from Analytic Jena (Jena, Germany). Coomassie Brilliant Blue R-250 was obtained from Thermo Scientific Pierce Protein Biology Products (Rockford, IL, USA), and polypeptide molecular weight markers for electrophoresis were purchased from Pharmacia (Uppsala, Sweden). All other chemicals and reagents used were of analytical grade and obtained from Avantor Performance Materials (Gliwice, Poland).

### 3.2. Protein Isolation

The flaxseed cake was ground using a laboratory mill and extracted thrice with *n*-hexane (1:4, *w*/*v*) at room temperature for 30 min to remove the remaining oil. The protein isolation from the defatted flour was conducted using alkaline extraction and precipitation at the isoelectric point according to the procedure described by Dev and Quensel [28] and modified with some adjustments by Udenigwe et al. [21]. Briefly, flaxseed flour was mixed thoroughly with water (1:20, *w*/*v*), and to reduce the viscosity of the suspension, its pH was adjusted to 5.0 with 2 M HCl. Cellulase was added at 10 mg/g of flour, and the mixture was heated at 37 °C in a water bath with shaking for 4 h. Subsequently, the pH of the mixture was adjusted to 9.5 using 2 M NaOH, and the proteins were extracted during incubation for 2 h at room temperature. The protein extract was separated from the pellet by centrifugation at 10 °C for 30 min at 4000× *g* (MPW-350, MPW Med. Instruments, Warsaw, Poland). The extraction of the proteins from the pellet was repeated once more. Supernatants from both extractions were combined, and the pH of the solution was adjusted to 4.0 using 0.2 M HCl. The precipitated proteins were separated from the supernatant by centrifugation at 10 °C for 30 min at 8700× *g*. The obtained pellet was suspended in a small volume of deionised water and neutralised (0.2 M NaOH). The protein suspension was placed in membrane tubes with a MW cut-off of 6–8 kDa (Spectra/Por, Spectrum Laboratories, Rancho Dominguez, CA, USA) and dialysed at 4 °C towards deionised water with a regular water change every 8 h. After 48 h, the content of the membrane tubes was lyophilised over approximately 48 h at −50 °C and 0.021 mbar (FreeZone 6 Liter Freeze Dry System, Labconco, Kansas City, MO, USA). The obtained protein isolate was stored at 4 °C.

### 3.3. Protein Hydrolysis

Next, 2 g portions of the protein isolate were suspended in deionised water at a ratio of 1:20 (*w*/*v*). The pH of each suspension was adjusted to the pH optimal for the enzyme used, i.e., 8.0 for Alcalase, 7.5 for trypsin and pancreatin, 7.0 for Flavourzyme and 6.5 for papain. The suspension was heated to 50 °C. When the temperature was reached, the enzyme, which was dissolved in 0.3 mL of water, was added at 15 mAU/g of protein (AU–Anson unit). The pH of the reaction mixtures was kept constant using an ETS 822 end point titration system working in the pH-stat mode (Radiometer Analytical, Copenhagen, Denmark). Then, 0.5 M NaOH was used as a titrant. The hydrolysis was terminated after exactly 120 min by heating at 100 °C for 5 min. The hydrolysates obtained were frozen and lyophilised.

### 3.4. Characteristics of Hydrolysates

#### 3.4.1. DH Determination

The DH was calculated based on the equation proposed by Adler-Nissen [29]:

(1) $$\mathrm{DH}=h/h_{tot}\;\times 100\%$$

where *h* is the number of peptide bonds cleaved during hydrolysis, and *h_tot_* is the total number of peptide bonds in the protein. The number of peptide bonds was quantified based on the determination of the content of free α-NH_2_ groups in the hydrolysate and the primary material using a spectrophotometric method with OPA and was expressed as meqv Leu/g protein [30]. The *h_tot_* was evaluated after 16 h of acidic hydrolysis (6 M HCl) of the proteins at 110 °C. The protein content of the hydrolysates was determined using the Kjeldahl method (*N* × 6.25) [31].

#### 3.4.2. Tricine-SDS-PAGE Separation

Electrophoretic separation of the protein isolate and hydrolysates was performed using a Bio-Rad Mini-Protean Tetra Cell system (Bio-Rad, Hercules, CA, USA) in the buffer and gel system proposed by Schagger and Jagow [32]. The separation was carried out using 4% stacking, 10% spacer and 16% resolving gels at voltages of 30, 50 or 80 V, respectively. The samples (2 mg/mL) were heated in reducing buffer (62.5 mM Tris-HCl, pH 6.8; 2.0% (*w*/*v*) SDS; 0.002% (*w*/*v*) bromophenol blue; 5% (*v*/*v*) β-mercaptoethanol; 10% (*v*/*v*) glycerol) at 100 °C for 5 min prior to analysis. A total of 0.2 μg of the sample was loaded per well. Protein bands were stained with Coomassie Brilliant Blue R-250. The MWs of the resolved proteins and polypeptides were estimated by the Quantity One Software (Bio-Rad) based on the separation of MW standards.

#### 3.4.3. MW Distribution by SE-HPLC

The SE-HPLC separation of the protein isolate and its hydrolysates was carried out according to a previously described method [10]. The Shimadzu HPLC system composed of a LC-10AD_Vp_ pump, a SPD-M10A_Vp_ photo-diode array detector, and a SCL-10A_Vp_ system controller (Kyoto, Japan) was applied. A 20 μL sample at a concentration of 5 mg/mL was injected onto a TSKgel G2000SW_XL_ column (7.8 × 300 mm, 5 μm, Tosoh Bioscience, Tokyo, Japan) and eluted with acetonitrile:water:trifluoroacetic acid (45:55:0.1 v/v/v) at a flow rate of 0.5 mL/min. Six standard molecular markers, albumin from chicken egg white (45 kDa), cytochrome C (12.4 kDa), aprotinin (6.5 kDa), angiotensin II acetate (1.046 kDa), leucine enkephalin (556 Da) and Val-Tyr-Val (379 Da), were used for column calibration.

### 3.5. Antioxidant Activity Assays

#### 3.5.1. Antiradical Activity towards ABTS^•+^

The antiradical activity of flaxseed protein hydrolysates towards ABTS^•+^ was evaluated using the method described by Re et al. [33]. ABTS radical cations were generated from ABTS mixed with a 2.45 mM potassium persulfate solution for 16 h. Directly before the analysis, the stock solution was diluted with water at a ratio of 1:50 (*v*/*v*). Solutions of the protein and the hydrolysates at 2 mg/mL were prepared in 0.1 M phosphate buffer, pH 7.0. A spectrophotometric assay was carried out in a 96-well plate using an Infinite M1000 microplate reader (Tecan, Männedorf, Switzerland). A total of 10 µL of isolate or hydrolysate solution was pipetted into the wells, and 200 µL of the ABTS^•+^ working solution warmed to 30 °C was added. In parallel, the reactions with Trolox in the range of concentration of 0.01–0.5 mmol/mL as a standard were carried out. The absorbance was read at λ = 734 nm after a 6 min reaction time. The results were expressed in mmol of Trolox equivalents per g of isolate or hydrolysate.

#### 3.5.2. PCL-ACL Assay

The scavenging activity of the protein isolate and its hydrolysates was evaluated by a PCL method [34], in which O_2_^•−^ were generated by the optical excitation of luminol. The analysis was carried out using ACL kits (Analytik Jena) and a Photochem device equipped with the PCLsoft software (Analytik Jena). Briefly, 2.3 mL of methanol (reagent 1), 200 μL of a buffer solution (reagent 2) and 25 μL of luminol (reagent 3) were mixed. Then, 10 μL of a methanolic solution containing the hydrolysate (0.1 mg) or standard (Trolox, 0.5–3.0 nmol) was added. The results were expressed in µmol of Trolox equivalents per g of sample.

#### 3.5.3. FRAP Assay

The FRAP assay was performed according to the method proposed by Benzie and Strain [35] with slight modification. The FRAP reagent was prepared by mixing 0.3 M acetate buffer with 10 mM TPTZ at pH 3.6 in 40 mM HCl and 20 mM FeCl_3_ × 6H_2_O pH 3.6, at the ratio of 5:1:1 (*v*/*v*/*v*), respectively. The isolate and hydrolysates were dissolved in water at 1 mg/mL. Solutions of FeSO_4_ × 7H_2_O in the range of concentrations of 0.03–0.9 μmol/mL were used for the calibration. The samples (40 μL) were placed into a 96-well plate, and 200 μL of the FRAP reagent heated to 37 °C was added. The reaction was carried out at 37 °C, and the absorbance was read at λ = 593 nm (Infinite M1000, Tecan). The results were expressed in mmol of Fe^2+^ per g of hydrolysates based on the calibration curve.

#### 3.5.4. Fe^2+^ Chelating Ability

The chelating ability of the flaxseed protein hydrolysates for ferrous ions was evaluated using the ferrozine method [36]. For this purpose, 200 μL portions of the isolate and hydrolysate solutions in water at 2 mg/mL were mixed with 20 μL of 0.4 mM FeCl_2_ × 4H_2_O. The amount of Fe^2+^ that was not bound by hydrolysates was determined spectrophotometrically by adding 40 μL of 5 mM ferrozine. The absorbance was measured after 10 min at λ = 562 nm using an Infinite M1000 microplate reader (Tecan). The percentage of bound Fe^2+^ was calculated.

### 3.6. Statistical Analysis

Flaxseed protein hydrolysis was carried out in triplicate for each enzyme. All the antioxidant tests were performed in triplicate at least. The measurements with their SD are depicted in the figures. The results of the DH and antioxidant activity were analysed by one-way analysis of variance (ANOVA) using the GraphPad Prism software (version 6.04 for Windows, GraphPad Software Inc., La Jolla, CA, USA). Tukey’s multiple comparison test was carried out to compare the values (*p* < 0.05).

## 4. Conclusions

The treatment of flaxseed proteins with proteases with high specificity (trypsin and papain) or enzymes with a broad range of action (pancreatin, Alcalase and Flavourzyme) produced hydrolysates with a high radical scavenging activity, ability to reduce ferric ions and ferrous ion chelation activity in comparison to the original proteins. Trypsin and papain degraded flaxseed proteins to a DH of 9.0% and 2.6%, respectively. Alcalase, Flavourzyme and pancreatin were more effective in cleaving the peptide bonds. However, the high DH did not warrant the high antioxidant activity of the hydrolysate. Generally, among the hydrolysates examined, those obtained using Alcalase and pancreatin were superior in terms of their antioxidant activity. Their DH values were 15.4% and 29.3%, respectively. The Flavourzyme hydrolysate with a DH of 48% quenched O_2_^•−^ and chelated Fe^2+^ ions to a lesser extent than the Alcalase and pancreatin hydrolysates. The present study shows that neither DH nor the MW distributions of the hydrolysates correlated with the antioxidant activity of the flaxseed protein hydrolysates obtained using different proteases. The pancreatin hydrolysate containing mainly peptides with a MW < 1 kDa was as active as the Alcalase hydrolysate, which contained larger constituents (<6.5 kDa).

Papain was the least effective in the release of peptides with antioxidant activity from flaxseed proteins among the enzymes applied. This hydrolysate was characterised by a low DH, and the content of polypeptides and peptides had a wide range of MWs.

It can be concluded that the type of enzyme used for the hydrolysis of flaxseed proteins determines the antioxidant activity of the respective hydrolysates.

## Abbreviations

DH	degree of hydrolysis
MW	molecular weight
SDS-PAGE	sodium dodecyl sulfate-polyacrylamide gel electrophoresis
SE-HPLC	size exclusion high-performance liquid chromatography
ABTS	[2,2′-azino-bis-(3-ethylbenzothiazoline-6-sulfonic acid)] diammonium salt
PCL	photochemiluminescence
ACL	antioxidant capacity of lipid-soluble substance
FRAP	ferric reducing antioxidant power
BHT	butylated hydroxytoluene
BHA	butylated hydroxyanisole
RP-HPLC	reversed phase high-performance liquid chromatography
DPPH	2,2-diphenyl-1-picrylhydrazyl
TPTZ	2,4,6-Tris(2-pyridyl)-*S*-triazine
EC	Enzyme Commission
OPA	*o*-phthaldialdehyde
AU	Anson unit
